# Nicotinamide Effectively Suppresses Fusarium Head Blight in Wheat Plants

**DOI:** 10.3390/ijms22062968

**Published:** 2021-03-15

**Authors:** Yasir Sidiq, Masataka Nakano, Yumi Mori, Takashi Yaeno, Makoto Kimura, Takumi Nishiuchi

**Affiliations:** 1 Division of Life Science, Graduate School of Natural Science and Technology, Kanazawa University, Kanazawa 920-1192, Japan; ys120@ums.ac.id; 2 Biology Education, Faculty of Teacher Training and Education, Universitas Muhammadiyah Surakarta, Sukoharjo 57162, Indonesia; 3 Institute for Gene Research, Advanced Science Research Center, Kanazawa University, Kanazawa 920-8640, Japan; masa4nak@gmail.com (M.N.); moriyumi0122@gmail.com (Y.M.); 4 Department of Agriculture, Ehime University, Ehime 790-8566, Japan; yaeno@agr.ehime-u.ac.jp; 5 Division of Molecular and Cellular Biology, Graduate School of Bioagricultural Sciences, Nagoya University, Nagoya 464-8601, Japan; mkimura@agr.nagoya-u.ac.jp

**Keywords:** nicotinamide, pyridine nucleotide, fusarium head blight, trichothecene mycotoxin, DNA hypomethylation, antifungal compound, metabolomics

## Abstract

Pyridine nucleotides such as a nicotinamide adenine dinucleotide (NAD) are known as plant defense activators. We previously reported that nicotinamide mononucleotide (NMN) enhanced disease resistance against fungal pathogen *Fusarium graminearum* in barley and Arabidopsis. In this study, we reveal that the pretreatment of nicotinamide (NIM), which does not contain nucleotides, effectively suppresses disease development of Fusarium Head Blight (FHB) in wheat plants. Correspondingly, deoxynivalenol (DON) mycotoxin accumulation was also significantly decreased by NIM pretreatment. A metabolome analysis showed that several antioxidant and antifungal compounds such as trigonelline were significantly accumulated in the NIM-pretreated spikes after inoculation of *F. graminearum*. In addition, some metabolites involved in the DNA hypomethylation were accumulated in the NIM-pretreated spikes. On the other hand, fungal metabolites DON and ergosterol peroxide were significantly reduced by the NIM pretreatment. Since NIM is relative stable and inexpensive compared with NMN and NAD, it may be more useful for the control of symptoms of FHB and DON accumulation in wheat and other crops.

## 1. Introduction

Plants defend themselves against various pathogens by both of chemical and physical defense systems [1]. Antimicrobial compounds which accumulate due to pathogen challenge are known as phytoalexins [2]. However, phytopathogens often overcome plant chemical defenses and enter the plant tissue. Therefore, biochemicals such as pesticides and fungicides are useful for the control of crop diseases [3,4]. However, the foods derived from agrochemical-contaminated crops often present health hazards to humans and domestic animals [5]. In addition, fungicide- and pesticide-resistant pathogen strains have been widely reported [6]. Plant defense activators induce the plant’s immune response. Probenazole is one such activator and can enhance resistance against rice blast fungus and other diseases [7,8]. Additionally, both 2,6-dichloroisonicotinic acid (INA) and benzo (1,2,3) thiadiazole-7-carbothionic acid S-methyl ester (BTH) are SA analogues which activate the systemic acquired resistance (SAR) signal transduction pathway [9,10].

Among the various phytopathogens, certain fungal pathogens produce toxic secondary metabolites (mycotoxins) which are harmful to humans and domestic animals [11,12]. *Fusarium* species such as *Fusarium graminearum* infect the flowers of wheat and barley spikes, and most of them can produce trichothecene mycotoxins [13,14]. These fusarium diseases are called Fusarium head blight (FHB). Commercial cultivars showing strong FHB resistance are not available in wheat and barley [15]. Therefore, fungicides are usually sprayed multiple times on their flowers [16]. However, pesticide-contaminated grains are also toxic to humans and animals. Therefore, in the present research, we attempt to identify useful natural products for the control of Fusarium head blight in cereal crops. In a previous study, we found that nicotinamide mononucleotide (NMN), a precursor of nicotinamide adenine dinucleotide (NAD), was highly accumulated in FHB-resistant barley cultivars [17]. We also revealed that NMN acted as a plant defense activator in Arabidopsis. Furthermore, the application of NMN was shown to enhance disease resistance against *F. graminearum* and suppress deoxynivalenol (DON) mycotoxin accumulation in barley [17]. We also found that l-Thr can suppress trichothecene biosynthesis in *F. graminearum* [18]. Thus, these metabolites may be useful for the control of disease injury and mycotoxin reduction in cereals.

Recently, extracellular nicotinamide adenine dinucleotide (eNAD) was shown to be able to activate plant immune response by binding to plant receptors [19,20]. Therefore, increasing the eNAD content was found to be effective in suppressing the disease resistance of Arabidopsis to *Pseudomonas syringae* pv. *maculicola* ES4326 (*Psm ES4326*) [21]. As stated above, NMN also acts as a plant defense activator and enhances FHB disease resistance in Arabidopsis and barley [17]. Since NAD and NMN contain the nucleotide, they are relatively unstable and expensive to produce. Among these derivatives, nicotinamide, which is stable and cheap, has antioxidant activity [22]. Therefore, herein, we examine the effects of nicotinamide on the suppression of FHB in wheat plants. For this purpose, we use a dwarf model wheat cultivar USU-Apogee that is susceptible to FHB. We reveal that the application of nicotinamide is effective in suppressing FHB in wheat plants. A metabolome analysis suggested that many defense-related metabolites were likely involved in the NIM-induced FHB resistance.

## 2. Results

### 2.1. Disease Development of FHB in the Wheat Model Cultivar

**Figure 1:**
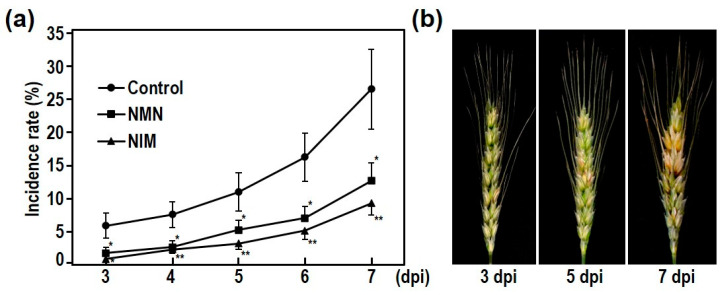
Disease incidence of Fusarium head blight (FHB) gradually increased from 3 dpi to 7 dpi. (**a**) The incidence of FHB disease on spikes of wheat cultivar USU-Apogee from 3 to 7 dpi. These spikes were sprayed with water, NMN, or NIM before inoculation. Each bar represents standard error (*n* = 12). Results of student’s t-test are shown: * *p* < 0.05, ** *p* < 0.01. (**b**) Representative photographs of symptom development in the water-treated control spikes at 3, 5, and 7 dpi.

Since the USU-Apogee is a dwarf wheat cultivar with a short life cycle, this cultivar is useful for the study of FHB in wheat [23]. USU-apogee revealed the FHB susceptible phenotype, which is similar to that of the known cultivar, Wheaton [23]. Therefore, we used the USU-apogee to evaluate the effects of NIM and NMN on FHB resistance. We monitored the spread of disease on the wheat spike after inoculation of *F. graminearum*. Fungal conidia were sprayed onto spikes with open flowers, and then inoculated spikes were kept in high humidity for two days. The disease symptoms appeared on the wheat spikes at 3 days postinoculation (dpi) in the control treatment. Figure 1a,b show that the symptoms of FHB disease became visible at 3 dpi in the flowerets of the control inoculated spikes. As shown in Figure 1a, the rate of incidence at 3 dpi was only 5%, but this gradually increased to 10% at 5 dpi in the control treatment. From 5 to 7 dpi, the rates of disease incidence increased rapidly to about 26% in the control inoculated spikes. As shown in Figure 1b, severe symptoms were observed only at 7 dpi in control spikes.

### 2.2. Nicotinamide Pretreatment Enhanced the FHB Resistance in Wheat Plants

In this study, we examined whether NMN and related metabolite NIM were effective in suppressing the disease symptoms of FHB and DON accumulation in wheat spikes. NIM does not contain any nucleotides, and is stable at room temperature. In addition, the cost of NIM is significantly less than those of NMN and NAD. For this purpose, solutions of NMN or NIM were sprayed onto the wheat spikes prior to inoculation of *F. graminearum*. As shown in Figure 1a, the incidence rates of inoculated spikes by NMN- and NIM-pretreatment decreased at 3 dpi in the water-pretreated control spikes. This fact indicated that plant immune response had already been activated by NMN- and NIM-pretreatment in the early stage of infection. Disease symptoms in the inoculated spikes after pretreatment with NMN and NIM gradually developed, and finally reached about 12% and 8%, respectively, at 7 dpi (Figure 1a,b). As stated above, the incidence rate in the water-pretreated spikes was about 26%. Significant differences of disease incidence were observed in NMN- and NIM-pretreated spikes compared with water-pretreated spikes; see Figure 1a. This result clearly showed that both NMN and NIM are capable of inducing plant defense response to suppress the development of FHB in wheat spikes. As previously reported, NMN and NAD are effective at decreasing the symptoms of FHB disease in barley and Arabidopsis. In addition, NMN and NIM application was also found to be useful in controlling FHB disease in wheat plants. As stated above, although NIM did not contain any nucleotides, significant suppression of FHB symptoms was observed in NIM-pretreated wheat spikes. Since NIM has higher stability with lower cost compared to pyridine nucleotides, NMN and NAD, it is potentially a more useful candidate for the control of FHB.

**Figure 2:**
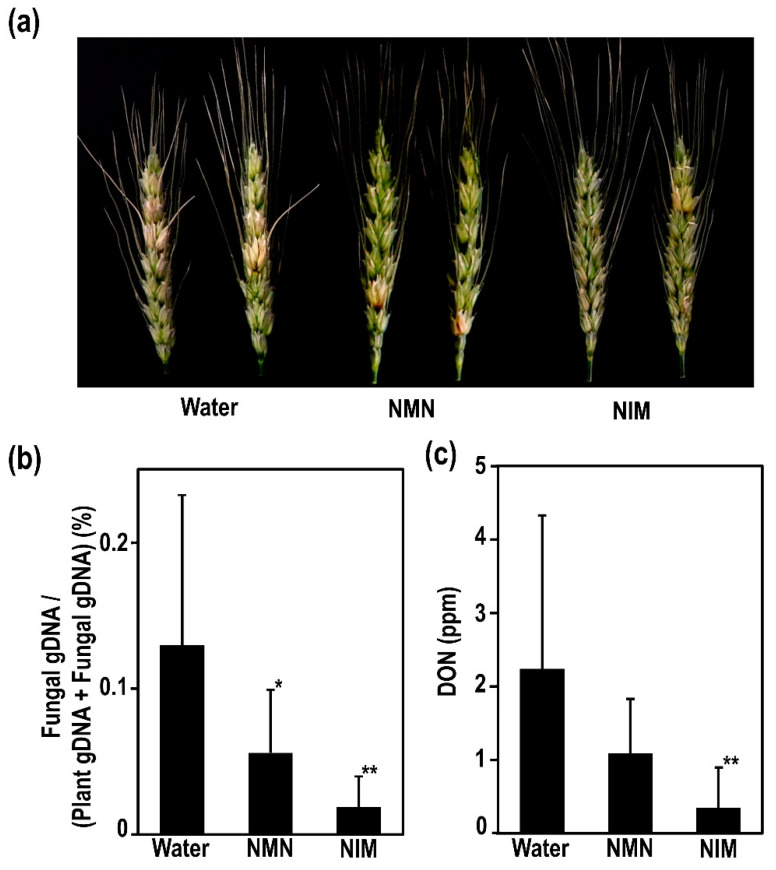
Nicotinamide mononucleotide (NMN) and nicotinamide (NIM) pretreatment enhanced disease resistance against *F. graminearum* in wheat spikes. NMN or NIM was sprayed onto spikes of susceptible cultivar USU-Apogee before inoculation. A conidia solution with 1x10^4^ conidia/mL was applied to these spikes by spraying. (**a**) The representative photographs of inoculated spikes at 7 dpi after water, NMN or NIM pretreatment. (**b**) The ratio of the fungal gDNA to the total gDNA in the water-, NMN-, NIM-pretreated spikes were quantified by qPCR, (**c**) DON accumulations of water-, NMN-, and NIM-pretreated spikes were measured. Each bar represents standard deviation (*n* = 12). Student’s *t*-test: * *p* < 0.05, ** *p* < 0.01.

To further analyze the effects of NMN and NIM in the control of FHB, fungal gDNA and DON accumulation in wheat spikes were measured. The DON accumulation was sometimes not correlated with the severity of disease symptoms in wheat and barley [24]. Therefore, the measurement of DON accumulation was necessary to evaluate the efficacy of the FHB control agents. Figure 2a shows that NMN and NIM pretreatment effectively suppressed the disease symptoms at 7 dpi in wheat spikes. The ratio of the fungal gDNA to the total gDNA in the control spikes was about 0.13%; however, those were apparently decreased to 0.06% and 0.02% in NMN- and NIM-pretreated wheat spikes, respectively; see Figure 2b. This result indicated that the NMN and NIM pretreatment effectively suppressed the propagation of cells of *F. graminearum* in wheat spikes. In the control, DON accumulated at more than 2 ppm at 7 dpi in the inoculated spikes; see Figure 2c. In contrast, the NMN and NIM-pretreated spike exhibited the about 1 ppm and 0.35 ppm DON accumulation, respectively. Thus, the propagation of pathogenic cells and DON accumulation were reduced to one-sixth and one-fifth by NIM pretreatment. The effects of NMN were similar, but slightly weaker, compared with NIM pretreatment. As mentioned, both NMN and NIM pretreatment effectively suppressed the initial infections by 3 dpi; see Figure 1a. Then, the increasing rates of disease incidence in the NMN- and NIM-pretreated spikes were also lower than those of the water-pretreated control spikes at 3 and 7 dpi. Therefore, the initial infection and development of FHB disease were effectively suppressed by NMN and NIM application. Long term incubation after inoculation is important for the evaluation of FHB severity. Therefore, we examined the incidence rates of FHB disease from 4 to 28 dpi in our inoculation system; see Figure S1. In our experimental condition, the incidence rates reached about 100% at 12 dpi. However, the effects of NIM and NMN were still observed at 12 dpi (Figure S2).

Furthermore, we examined the effects of NMN and NIM on two other Japanese cultivars; see Figures S3 and S4. It is known that the FHB resistance of Harukirari and Haruyutaka are intermediate and weak, respectively, among Japanese varieties [25,26]. The effects of NIM and NMN were also observed in two cultivars, although different degrees of FHB resistance were observed among the two cultivars (Figures S3 and S4). These results suggested that NIM and NMN may be useful to control FHB disease in many wheat cultivars.

### 2.3. Effects of NMN and NIM Pretreatment on Pyridine Metabolites of Wheat Inoculated Spikes

**Figure 3:**
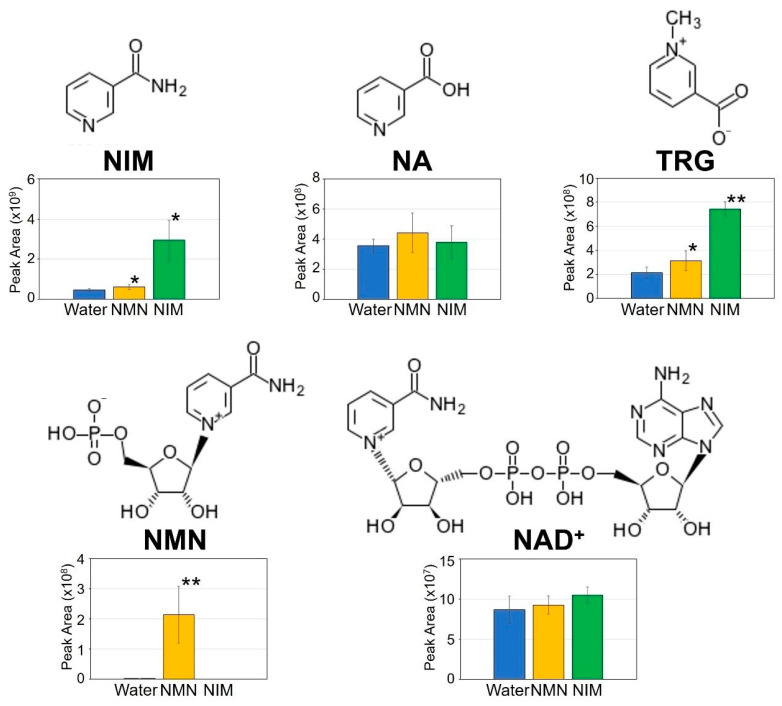
Effects of nicotinamide mononucleotide (NMN) and nicotinamide (NIM) pretreatment on pyridine metabolites in the wheat inoculated spikes. Relative amounts of metabolites NMN, NIM, nicotinic acid (NA), trigonelline (TRG), and nicotinamide adenine dinucleotide (NAD^+^) were measured in water-, NMN-, and NIM-pretreated spikes based on the peak area of their precursor ions. Each bar represents standard deviation. Student’s *t*-test: * *p* < 0.05, ** *p* < 0.01, *n* = 4–5.

As mentioned above, disease symptoms and DON accumulations were suppressed by NIM and NMN pretreatment in wheat spikes. To examine the effects of these metabolites, we investigated the amounts of NMN, NIM and related metabolites using LC-MS/MS. We extracted the metabolites from inoculated spikes at 7 dpi with water, NMN, and NIM pretreatment. These extracted solutions were separated by UPLC and analyzed by MS spectrometer (Orbitrap QE plus). As shown in Figure 3, we measured pyridine nucleotides and related metabolites in these samples. The content of NMN was still significantly accumulated in the inoculated spikes after seven days of NMN spraying. In contrast, the NMN content was very low in the NIM- and water-pretreated leaves. This result showed that NMN was still present after seven days of spraying and likely affected the defense responses of wheat spikes. The contents of NIM and trigonelline (TRG) were slightly elevated in the NMN-pretreated spikes. The NAD and nicotinic acid (NA) contents in NMN-pretreated spikes were not different from those of the water-pretreated spikes. The apparent increase by NMN pretreatment was observed only in NMN content among five metabolites. NIM pretreatment caused a significant increase in not only NIM, but also TRG. Since TRG is a pyridine alkaloid which acts as an antimicrobial compound, accumulation of TRG likely contributed the enhanced FHB resistance in wheat spikes.

### 2.4. Differentially Accumulated Metabolites in the NMN- and NIM-Pretreated Spikes

**Figure 4:**
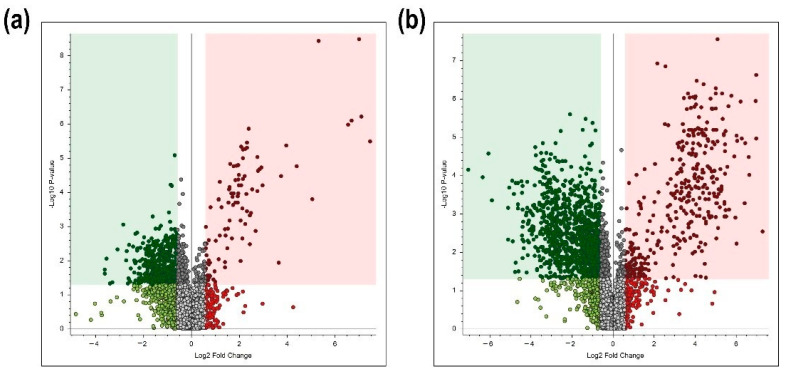
Volcano plots illustrate the distributions of quantified metabolites in the nicotinamide mononucleotide (NMN)- and nicotinamide (NIM)-pretreated spikes at 7 dpi. The results of (**a**) NMN- and (**b**) NIM-pretreated spikes are shown. The vertical-axis is the −log10 of the *p*-value and the horizontal-axis is log2 of fold change of NMN- or NIM-pretreated spikes compared to the control ones. Cutoff values of fold change and *p*-value were 1.5 and 0.05, respectively. The dots indicate detected metabolites containing the unidentified and redundant ones. Red squares indicate significantly accumulated metabolites, while green squares show significantly reduced metabolites.

We also performed comparative metabolome studies on water-, NMN-, and NIM-pretreated spikes at 7 dpi. Many differentially accumulated metabolites were identified by LC-MS/MS analysis. In Figure 4, volcano plots show differentially accumulated metabolites by NMN and NIM pretreatment. These metabolites contained unspecified and redundant ones. The red area indicates upregulated metabolites due to NMN or NIM pretreatments (fold change > 1.5 and *p* value < 0.05); the green area shows downregulated metabolites due to NMN or NIM pretreatment (Fold change < 0.67 and *p* value < 0.05). It was found that 99 and 486 metabolites were up- and down-regulated, respectively, by NMN pretreatment in inoculated spikes at 7dpi. On the other hand, NIM pretreatment induced and reduced the contents of 375 and 1174 metabolites, respectively, in inoculated spikes. Thus, the effects of NIM pretreatment on the metabolite profile were apparently greater than those of NMN pretreatment.

**Figure 5:**
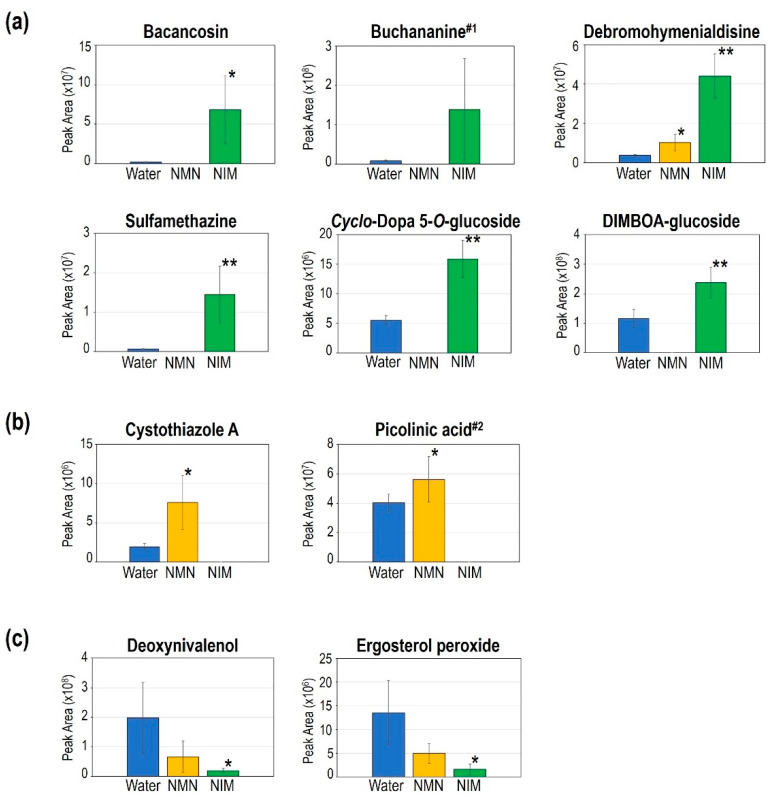
Representative differentially regulated metabolites by nicotinamide mononucleotide (NMN) and nicotinamide (NIM) pretreatment in inoculated spikes at 7 dpi. (**a**) Metabolites significantly accumulated by NIM pretreatment, (**b**) metabolites significantly accumulated by NMN pretreatment, and (**c**) metabolites significantly reduced by NIM pretreatment. The peak areas of the vertical axis show the abundance of each metabolite. (1) Buchananine was more than 1.5-fold more accumulated without statistical significance in NIM-pretreated spikes. (2) Picolinic acid was significantly accumulated in the NMN-pretreated spikes with a less than 1.5-fold change. Each bar represents one standard deviation (*n* = 4–5). Student’s *t*-test: * *p* < 0.05, ** *p* < 0.01.

Next, we checked the properties of each differentially regulated metabolite by NMN or NIM pretreatment. These metabolites were classified into three groups: (a) highly accumulated in the NIM-treated spikes; (b) highly accumulated in the NMN-pretreated spikes; and (c) reduced metabolites in the NMN- and NIM-pretreated spikes. Figure 5 shows the representative metabolites in each group. Antibiotics, as noted in Figure 5a, were highly accumulated in the NIM-pretreated spikes after inoculation of *F. graminearum*. Bacancosin is a plant saponin, i.e., a natural detergent which is harmful to the membranes of microbials [27]. Both debromohymenialdisine and buchananine are classified as alkaloids, most of which have antimicrobial activities [28]. It has been reported that buchananine is an antifungal compound [29], although large variation was observed in its content in NIM-pretreated spikes. Sulfamethazine has also been reported as an antimicrobial compound [30]. On the other hand, *cyclo*-Dopa 5-*O*-glucoside may act as a ROS scavenger [31]. DIMBOA-glucoside is likely related to defense signaling [32]. As shown in Figure 5b, cystothiazole A and picolinic acid are specifically accumulated by NMN-pretreatment. It has been reported that cystothiazole A has antifungal activity to *Phytophthora capsica* [33]. The fold increase of picolinic acid content by NMN-pretreatment was not large, but was statistically significant. Picolinic acid is known as an inducer of plant defense response against *Magnaporthe oryzae* [34].

As shown in Figure 5c, two fungal metabolites are reduced by NMN- and NIM-pretreatment. As stated above, DON was significantly decreased in the NIM-pretreated spikes. Correspondingly, derivatives of ergosterol, which is specifically found in the fungal membrane, were also shown to be significantly decreased in the NIM-pretreated spikes. These results also support the hypothesis that FHB disease and mycotoxin accumulation can be significantly suppressed by NIM pretreatment.

## 3. Discussion

Many natural products have been put forward as candidates to control FHB resistance and mycotoxin accumulation in wheat and barley. Some antifungal compounds have been proposed as potential FHB control agents. The plant alkaloid antofine was shown to have antifungal activity, and significantly suppressed the development of *F. graminearum* at 150 ug/mL concentration in wheat spikes [35]. In this study, we also identified three alkaloids, TRG, buchananine, and debromohymenialdisine, that were highly accumulated in the NIM-pretreated spikes in comparison with control spikes. Since both TRG and buchanaine are pyridine alkaloids, the application of NIM likely contributed to their accumulation. It has been reported that high nitrogen causes TRG accumulation in tomato leaves and enhances resistance against the fungal pathogen, *Fusarium oxysporum* [36]. In addition, the application of TRG to the leaves of barley plants reduced disease symptoms due to powdery mildew up to 56% [37]. Buchananine was shown to possess broad antibacterial activity against eight out of the ten bacteria species examined [38]. Therefore, these metabolites likely play roles in FHB resistance in wheat plants. 

Debromohymenialdisine was reported as an effective insecticidal compounds [39]. As stated above, bacancosin is a plant saponin which is harmful to microbial membranes [27]. Sulfamethazine also has antimicrobial activities [30]. Thus, the accumulation of these antimicrobial compounds in NIM likely plays an important role in enhancing FHB disease resistance in wheat spikes.

It is known that NIM has antioxidant activity relative to abiotic stress in plants [22,40]. Similarly, we previously reported that NMN pretreatment decreased the ROS accumulation in Arabidopsis leaves inoculated with *F. graminearum* [17]. On the other hand, DNA hypomethylation effects by NIM have been reported in plant cells [22]. It has also been reported that TRG application reduces DNA methylation in barley and enhances disease resistance to powdery mildew [37]. In general, DNA hypomethylation is related to the activation of gene expression [41]. Furthermore, sulfamethazine, which is a pyrimidine, suppresses epigenetic silencing through DNA methylation [42]. Thus, these metabolites, including NIM itself, likely cause DNA hypomethylation and the activation of immune response genes. DIMBOA-glucoside has been reported as an anti-insect compound in plants [43]. In addition, DIMBOA-glucoside was found to be accumulated in the MPK6-overexpressed of *Zea mays* [44], suggesting that production of DIMBOA-glucoside was regulated by the MAPK-dependent defense signaling pathway. Interestingly, it has been reported that DIMBOA suppressed the expression of trichothecene biosynthetic genes in *F. graminearum* [45]. Mycotoxin production was shown to be significantly suppressed without disturbing fungal growth [45]. In this study, we did not detect DIMBOA in all wheat samples (data not shown). Thus, NIM and other metabolites have antioxidant and DNA hypomethylation activities, and these activities were likely involved in the observed enhanced FHB resistance in wheat spikes. However, we should experimentally confirm the occurrence of hypomethylation by NIM treatment in a future study.

As shown in Figure 5b, two metabolites were specifically accumulated in the NMN-pretreated spikes. Cystothiazole A has antifungal activity against a broad range of microorganisms including *Aspergillus fumigatus*, *Botritys cinerea* and *Phytophora capsici* [33]. Cystothiazole A is a bithiazole which inhibits respiration, since it interferes with NADH oxidation [33]. The application of picolinic acid at low concentrations stimulates antioxidative response, resulting in a decrease of blast symptoms in rice [34]. Therefore, these metabolites are at least partially involved in FHB resistance due to NMN.

We also identified two fungal metabolites which were significantly decreased in the NIM-pretreated spikes (Figure 5c). Such differences were also observed in the NMN-pretreated spikes, but there were not significant. A significant decrease in DON accumulation was confirmed in NIM-pretreated spikes by LC-MS/MS analysis (Figure 2c and Figure 5c). Ergosterol peroxide is a derivative of ergosterol that is specifically observed in the membrane of fungal cells [46]. A significant reduction of ergosterol peroxide was observed in the NIM-pretreated spikes, indicating that the amounts of fungal cells therein were apparently decreased (Figure 5c). This result is consistent with the quantification of fungal gDNA (Figure 2c). Taken together, NIM pretreatment effectively suppressed the progression of fungal cells and the production of mycotoxin in inoculated spikes. Our identified metabolites support the hypothesis that NIM is a useful candidate to control FHB disease in wheat and other crops.

Other natural FHB control agents have been reported. For example, tannic acid is a plant polyphenol which inhibits conidia germination and mycelium growth of *F. graminearum* [47]. The application of tannic acid was found to reduce disease severity and DON in spikes of wheat cultivar USU-Apogee [47]. Similar effects were observed in a field test with artificial inoculation of *F. graminearum* [47]. Recently, it was reported that the application of chitosan hydrochloride derived from chitin suppressed the severity of FHB and DON accumulation in wheat spikes. In addition, chitosan treatment activated the SAR signaling pathway and caused induction of some defense genes in wheat spikes [48]. Additionally, some natural products have been identified as inhibitors of trichothecene biosynthesis in *F. graminearum*. The glutamine analogue, acivicin, suppressed the expression of *Tri4*, *Tri5*, and *Tri6* genes and reduced mycotoxin production in the medium [49]. In addition, the amino acid l-Thr also suppressed trichothecene mycotoxin production in the medium and host plants [18]. Since these inhibitors of trichothecene biosynthesis exhibited different effects compared to NIM and NMN, additive effects can be expected, which may be useful for controlling FHB disease symptoms and mycotoxin accumulation in wheat spikes. Therefore, we will perform field inoculation tests using such mixtures in the near future.

## 4. Materials and Methods

### 4.1. Plant Material and Growth Conditions

Wheat plants (*Triticum aestivum)* cultivar USU-Apogee were used in this study [23]. The seeds were sown on filter paper supplied with water for two days in constant darkness at 22 °C. The germinated seedlings were then transferred to soil and grown in a growth chamber at 22 °C in a 16h light/8h dark illumination cycle (about 35,000 lux).

### 4.2. Pretreatment of Compounds and Fungal Inoculations

*Fusarium graminearum* strain H3 was used for the inoculation assay [17]. Fungal stocks were cultured on a potato dextrose agar (PDA) plate and stored at 4 °C. We prepared the conidia solution as previously described [50].

Pretreatments of chemicals were performed three days and 4 h before inoculation, as described previously [17]. NMN (Chombi-block Inc., San Diego, USA) and NIM (TCI Co., Ltd., Tokyo, Japan) was dissolved with sterile distilled water at a concentration of 10 mg/mL (10,000 ppm) and kept at −20 °C. The working concentration was 500 ppm with 0.01% (*v*/*v*) silwet L77. Twelve plants were used for each chemical treatment.

Spray inoculations with *F. graminearum* were performed using flowering wheat spikes. The concentration of conidia solution was adjusted to 1 × 10^4^ conidia/mL in 1x PBS with 0.001% (*v*/*v*) silwet L77, and was then applied to wheat spikes by spraying [17]. Inoculated wheat spikes were placed in a container with water and covered using plastic wrap to maintain high humidity. Then, inoculated spikes were incubated at 22 °C with low light intensity. At 2 dpi, the plastic wraps were removed. The inoculated spikes were incubated for an additional five days (total seven days after inoculation).

### 4.3. Evaluation of Disease Incidence Rates

The effects of NMN and NIM on FHB resistance were evaluated by the rate of disease incidence in the inoculated wheat spikes. The rate of disease incidence showed the ratio of the diseased florets to total florets in each spike [23]. Brown or premature bleaching in the single floret was counted as evidence of disease [51].

### 4.4. The Quantification of Fungal gDNA

Inoculated wheat spikes were crushed to obtain a fine powder using stainless beads and shake master neo (Bio Med. Sci., Tokyo, Japan) at 1500 rpm for 2 min with liquid nitrogen cooling. The genomic DNAs were isolated from about 70 mg fine powder using a Maxwell RSC Purefood GMO and Authentication Kit (Promega, Madison, WI, USA) with Maxwell RSC instrument (Promega, Madison, WI, USA). The quantities of *F. graminearum* gDNA and wheat gDNA in inoculated spikes were determined by qPCR using a AriaMx Real-Time PCR system (Agilent Tech., Santa Clara, CA, USA) with the reagent of 2× Brilliant III Ultrafast SYBR Green (Agilent Tech., Cedar Creek, TX, USA). For the quantification of *F. graminearum* gDNA, the *F. graminearum EF-1a* gene was amplified [17]. To quantify wheat gDNA, *T. aestivum α-tubulin* gene was amplified, as previously described [52].

### 4.5. Quantification of DON Accumulation

DON accumulation in wheat spikes was measured from 10 mg of fine powders using QuickScan DON3 (Envirologix, Inc., Portland, ME, USA). Five hundred microliters of sterile water was added to the fined powders. This were then vortexed at room temperature for one minute. Supernatants of samples were collected by centrifugation (5000 rpm) at room temperature for 10 min. The DON accumulation in 200 µL supernatant was measured using Envirologix QuickScan System (Envirologix, Inc., Portland, ME, USA) following the manufacturer’s instructions.

### 4.6. Extraction of Metabolites

The extraction of metabolites was conducted as previously described [53]. Briefly, 50 mg samples were measured and resuspended by vortex with 250 µL of precooled 80% (*v*/*v*) methanol containing 0.1% (*v*/*v*) formic acid. The samples were chilled on ice for 5 min and then centrifuged at 15,000 rpm for 5 min at 4 °C. Two hundred microliters of the supernatants was transferred to new tubes and diluted to a 53% (*v*/*v*) methanol concentration using water. Subsequently, the samples were centrifuged at 15,000 rpm at 4 °C for 10 min. The supernatants were transferred to vials for UHPLC.

### 4.7. UHPLC-MS/MS Analysis

An Ultimate 3000 UHPLC system (Thermo Fisher Scientific, San Jose, CA, USA) coupled with Orbitrap Q Exactive Plus (Thermo Fisher Scientific, San Jose, CA, USA) was used to analyze the metabolites. The separation of metabolites using UHPLC conditions was as follows: mobile phase A contained 0.1% (*v*/*v*) Formic acid and 5 mM ammonium acetate at pH 9.0 in water, while mobile phase B comprised 99.7% methanol. The extraction solutions were injected into the reverse phase column, Hypersil GoldColumn (100 × 2.1 mm with 1.9 µm particle size). The injection volume was 10 µ, the flow rate was 0.25 mL/min, and the column temperature was 20 °C. The gradient conditions were as follows: 0 min, 98% A and 2% B; 1.5 min, 98% A and 2% B; 12 min, 0% A and 100% B; 14 min 0% A and 100% B; 14.1 min 98% A and 2% B; 17 min 98% A and 2% B. The Orbitrap Q Exactive Plus mass spectrometer was operated in positive polarity mode with 3.2 kV of spray voltage. The capillary temperature was 320 °C; the temperature of the autosampler was 10 °C; 35 arb and 10 arb were the sheath gas flow rate and auxiliary gas flow rate, respectively [53].

### 4.8. Analysis of Metabolome Data

Compound discoverer v.3.1 (CD 3.1, Thermo Scientific, San Jose, CA, USA) was used for mass spectrometry data acquisition [53]. The settings were as follows: 5 ppm mass tolerance, 30 signal of intensity tolerance, and 1,000,000 of minimum peak intensity [54]. The annotations of metabolites were generated using ChemSpider (http://www.chemspider.com/) (accessed date: 29 January 2021) database, which consisted of integrating data from the Aracyc, Biocyc, KEGG pathways, mzVault, and mzCloud databases. Differentially accumulated metabolites by chemical treatments were selected with *p*-value < 0.05, fold change ≥ 1.5 and ≤ 0.667. Volcano plots were generated to show the distribution of differentially accumulated metabolites.

## Data Availability

The data presented in this study are available online within this article or supplementary materials.

## Supplementary Materials

The following are available online at https://www.mdpi.com/1422-0067/22/6/2968/s1, Figure S1: Incidence rates of FHB disease in wheat cultivar USU-Apogee from 4 to 28 dpi, Figure S2: FHB disease of wheat spikes was suppressed by NMN and NIM pretreatment at 12 dpi, Figure S3: NIM and NMN effectively suppressed FHB disease in wheat spikes cultivar Haruyutaka at 5 dpi, Figure S4: FHB disease was suppressed by NIM- or NMN-pretreatment in wheat cultivar Harukirari at 10 dpi.
