# Supplementary material for: Nicotinamide Effectively Suppresses Fusarium Head Blight in Wheat Plants
Source: Int J Mol Sci. 2021 Mar 15;22(6):2968. doi: 10.3390/ijms22062968 (PMC8000930; doi:10.3390/ijms22062968)
Supplement: Supplementary file 1 [file ijms-22-02968-s001.pdf]

International Journal of
Molecular Sciences

MDPI

Supplementary Figures

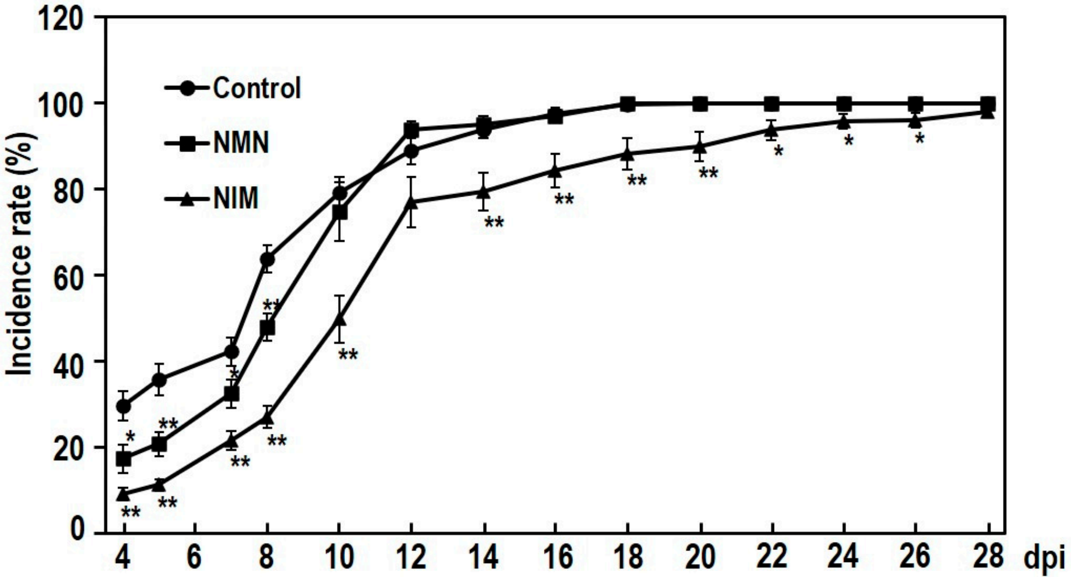

Figure S1. Incidence rates of FHB disease in wheat cultivar USU-Apogee from 4 to 28 dpi**.** In our experimental condition, disease incidence reached about 100% in control spikes at 12 dpi. However, incidence rates of FHB disease were significantly suppressed by NIM pretreatment at 26 dpi. Each bar represents standard error. Students' t-test: *p<0.05, **p<0.01, n=12

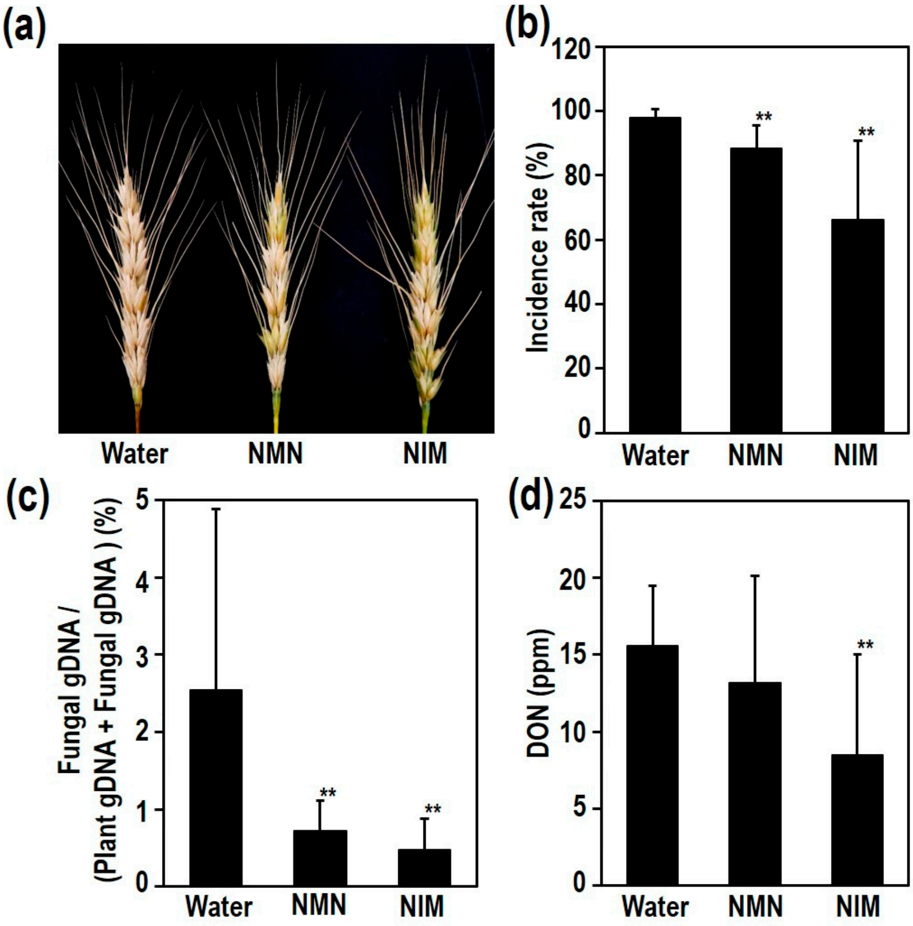

Figure S2. FHB disease of wheat spikes was suppressed by NMN and NIM pretreatment at 12 dpi. (a) Representative photograph of spikes pretreated by water, NMN, or NIM. (b) The incidence rates of FHB disease at 12 dpi. (c) Fungal gDNA of these spikes was quantified by qPCR. (d) DON contamination in these spikes was measured. Each bar represents standard deviation, students' t-test: ***p*<0.01, n=12.

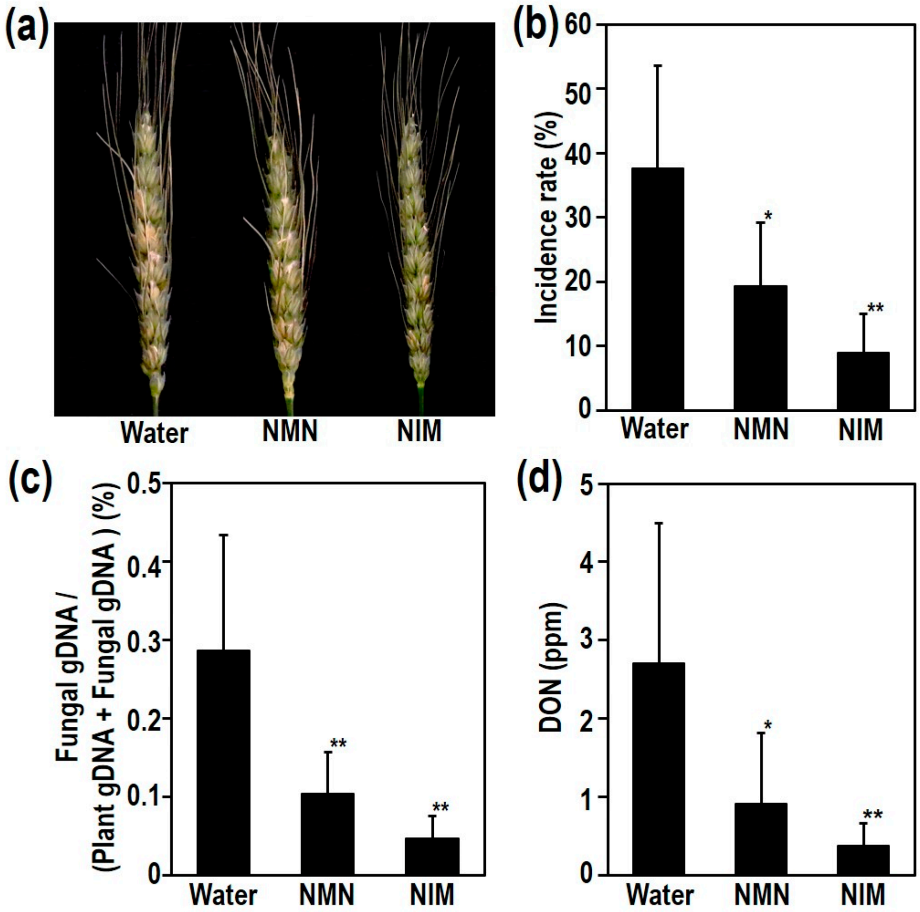

Figure S3. NIM and NMN effectively suppressed FHB disease in wheat spikes cultivar Haruyutaka at 5 dpi. (a) The representative images of water-, NIM- or NMN-pretreated spikes of wheat cultivar Haruyutaka at 5 dpi. (b) The disease incidence rate of FHB in these spikes. (c) Fungal gDNA was significantly decreased by NMN and NIM pretreatment. (d) DON accumulation was suppressed in NMN- or NIM-pretreated spikes compared with water-pretreated spikes. Each bar represents standard deviation, students' t-test: *$p<0.05$, **$p<0.01$, n=8.

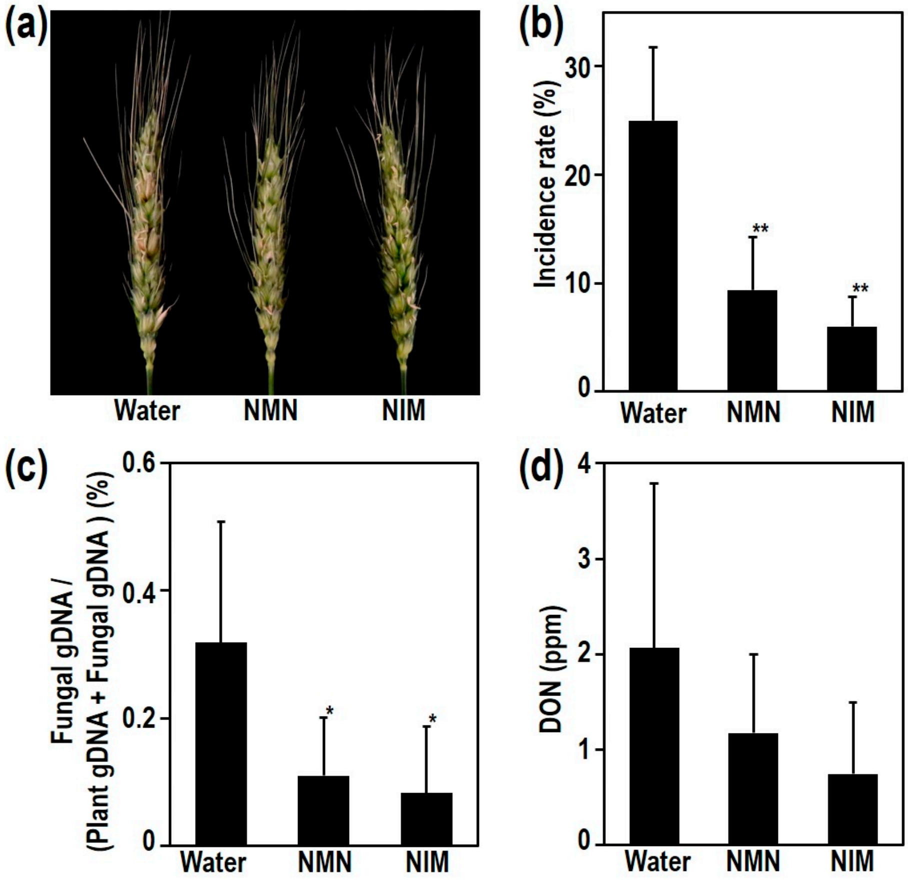

Figure S4. FHB disease was suppressed by NIM- or NMN-pretreatment in wheat cultivar Harukirari at 10 dpi. (a) Representative photograph of spikes pretreated by water, NMN, or NIM. (b) The incidence rates of FHB disease at 10 dpi. (c) Fungal gDNA of these spikes was quantified by qPCR. (d) DON contamination in these spikes was measured. Each bar represents standard deviation, students' t-test: *p<0.05, **p<0.01, n=8.
